# Mitochondrial Genomes from Two Specialized Subfamilies of Reduviidae (Insecta: Hemiptera) Reveal Novel Gene Rearrangements of True Bugs

**DOI:** 10.3390/genes12081134

**Published:** 2021-07-26

**Authors:** Fei Ye, Hu Li, Qiang Xie

**Affiliations:** 1 Department of Ecology and Evolution, School of Life Sciences, Sun Yat-sen University, Guangzhou 510275, China; yefei6@mail2.sysu.edu.cn; 2 State Key Laboratory of Biocontrol, Sun Yat-sen University, Guangzhou 510275, China; 3 Department of Entomology and MOA Key Lab of Pest Monitoring and Green Management, College of Plant Protection, China Agricultural University, Beijing 100193, China

**Keywords:** Heteroptera, Reduviidae, mitochondrial genome, gene rearrangement, phylogeny

## Abstract

Reduviidae, a hyper-diverse family, comprise 25 subfamilies with nearly 7000 species and include many natural enemies of crop pests and vectors of human disease. To date, 75 mitochondrial genomes (mitogenomes) of assassin bugs from only 11 subfamilies have been reported. The limited sampling of mitogenome at higher categories hinders a deep understanding of mitogenome evolution and reduviid phylogeny. In this study, the first mitogenomes of Holoptilinae (*Ptilocnemus lemur*) and Emesinae (*Ischnobaenella hainana*) were sequenced. Two novel gene orders were detected in the newly sequenced mitogenomes. Combined 421 heteropteran mitogenomes, we identified 21 different gene orders and six gene rearrangement units located in three gene blocks. Comparative analyses of the diversity of gene order for each unit reveal that the tRNA gene cluster *trnI*-*trnQ*-*trnM* is the hotspot of heteropteran gene rearrangement. Furthermore, combined analyses of the gene rearrangement richness of each unit and the whole mitogenome among heteropteran lineages confirm Reduviidae as a ‘hot-spot group’ of gene rearrangement in Heteroptera. The phylogenetic analyses corroborate the current view of phylogenetic relationships between basal groups of Reduviidae with high support values. Our study provides deeper insights into the evolution of mitochondrial gene arrangement in Heteroptera and the early divergence of reduviids.

## 1. Introduction

Mitochondria, the cell’s powerhouse, play an important role in energy metabolism and apoptosis [1]. As a semiautonomous organelle, mitochondrion has bacteria-like protein synthesis machinery and its own genome. In general, the typical insect mitochondrial genome (mitogenome) is a double-stranded circular molecule of 14–20 kb and encodes 13 proteins—essential components of the electron transfer chain and ATP synthase, two ribosomal RNAs (rRNAs), and 22 transfer RNAs (tRNAs) [2,3]. Although genome size and structure are highly conserved in most insect mitogenomes, various gene orders, and even changes in gene content and copy number indicate the plasticity and evolutionary history of mitogenome. Within Insecta, most clades retain the putative ancestral pancrustacean gene order [2,4], while a great variety of gene rearrangements have been reported in many lineages such as Psocodea [5,6], Thysanoptera [7,8], and Hymenoptera [9,10], which provides useful information to explore the evolutionary dynamics of rearrangement and deepens the understanding of the evolutionary pattern of insect mitogenomes.

Within hemipteran mitogenomes, gene rearrangements are mainly found in Sternorrhyncha (Aleyrodidae [11], Aphididae [12], Coccoidea [13]), Auchenorrhyncha (Delphacidae [14]), and Heteroptera (Enicocephalidae [15], Reduviidae [16,17,18], Pachynomidae [19], Aradidae [20], Pyrrhocoroidea [21]). Among which, Heteroptera (especially Reduviidae) display diverse rearrangement types including gene translocation, gene duplication, and gene loss. Assassin bugs (Reduviidae), the biggest predatory family of Hemiptera [22], comprise about 981 genera and nearly 7000 species in 24 subfamilies *sensu* Schuh and Weirauch, 2020 [23], or 25 subfamilies *sensu* Weirauch et al., 2014 [24]. To date, reduviid mitogenomes have been determined from only 11 subfamilies, and however many specialized lineages that have no mitogenomic data, such as Holoptilinae and Emesinae. Holoptilinae, often called feather-legged bugs with dense vestiture on body and appendages, contain 16 extant genera and about 80 species, which mainly occur in the southern Palearctic region, Old World tropics, and Australia [23]. Emesinae, thread-legged bugs, comprise about 90 genera and more than 900 species with elongated and slender bodies and long thread-thin legs, which ensconce themselves in dense vegetation, tree trunks, leaf litter, and spider webs [23]. A broadening survey of Reduviidae mitogenomes with higher taxon coverage, particularly at the subfamily and/or tribe levels, will be helpful to investigate the evolutionary pattern of heteropteran mitogenomes by a comparative genomics approach.

In the present study, we sequenced the first mitogenomes of Holoptilinae (*Ptilocnemus lemur*) and Emesinae (*Ischnobaenella hainana*), and detected two novel gene rearrangements, namely *trnI*-*trnW*-*trnQ*-*trnM*-*ND2*-*trnC* (*P. lemur*) and *ND2*-*trnW*-*trnC*-*COI*-//-*rrnL*-*rrnS*-Control region-*trnV* (*I. hainana*). The possible mechanism of both gene rearrangements was also inferred. Furthermore, based on the investigation of all the reported mitochondrial gene arrangements of true bugs, we explored the hotspot, mechanism, and evolutionary pattern of gene rearrangement in heteropteran mitogenomes and uncovered that Reduviidae greatly enhanced the gene arrangement diversity of heteropteran mitogenomes with independent evolution.

## 2. Materials and Methods

### 2.1. Assassin Bugs Collection and DNA Extraction

The specimens of *P. lemur* and *I. hainana* were collected at Kosciuszko National Park, New South Wales, Australia and Wuzhishan National Nature Reserve, Hainan, China, respectively, and then preserved in 100% ethanol under −20 °C. Total genomic DNA was extracted from the head and thorax of a single specimen using a DNeasy Blood and Tissue Kit (Qiagen, Hilden, Germany).

### 2.2. Genome Sequencing, Assembly and Analyses

Two DNA libraries were prepared with a 250-bp insert size and, subsequently, sequenced with a 150-bp paired end using the HiSeq X Ten platform at BGI Genomics (Shenzhen, China). After removing the adaptor contamination and low-quality sequences, 20,901,436 (*P. lemur*) and 26,845,556 (*I. hainana*) clean reads were used in *de novo* assembly using SOAPdenovo2 [25]. Finally, the corresponding mitogenome assemblies for both species were identified using BLAST [26] against the local database of Heteroptera mitogenomes. A total of 21,539 and 37,607 reads were assembled into *P. lemur* and *I. hainana* mitogenomes with an average coverage depth of 211× and 252×, respectively.

We used MITOS [27] to annotate newly sequenced mitogenomes, while, in order to improve the accuracy of annotation, we also re-confirmed the boundary of protein-coding genes (PCGs) and rRNA genes by an alignment with homologous genes from published heteropteran mitogenomes. Then, these two mitogenomes were deposited into GenBank under the accession numbers MW540749 (*P. lemur*) and MW619686 (*I. hainana*).

Base composition and codon usage were calculated using MEGA X [28], and base compositional skews were measured using the formulae AT-skew = (A − T)/(A + T) and GC−skew = (G − C)/(G + C) [29]. In order to evaluate the evolutionary rate of reduviid mitochondrial PCGs, we selected one species from each of the 13 subfamilies (Table S1) to calculate the rate of non-synonymous substitutions (Ka) and synonymous substitutions (Ks), and the ratio of Ka/Ks for each PCG using DnaSP 6 [30]. Moreover, to uncover the distribution pattern of mitochondrial gene rearrangement and identify the rearrangement hotspot of heteropteran mitogenomes, we summarized all 423 available heteropteran mitogenomes available in GenBank, including two newly sequenced mitogenomes, and defined six rearrangement units organized in three blocks. Furthermore, the family level gene rearrangement richness of each unit and the whole mitogenome, namely the ratio of the total number of different rearrangements of each unit in one family to the total number of all rearrangements of the corresponding unit in Heteroptera, was calculated to assess the diversity and taxon preference of mitochondrial gene rearrangement.

### 2.3. Phylogenetic Analyses

Phylogenetic relationships within Reduviidae were reconstructed based on mitochondrial PCGs, and mitochondrial (12S and 16S) and nuclear (18S and 28S) rDNA sequences using Bayesian inference (BI) and maximum likelihood (ML) methods. A total of 33 assassin bugs were selected as ingroups, and an additional three species from Pachynomidae, Miridae, and Nabidae were used as outgroups. The sampling details and GenBank accession numbers are shown in Table S2.

The nucleotide sequences of 13 PCGs were aligned based on amino acid alignments using MUSCLE [31] implemented in MEGA X. Four rDNA sequences were also aligned using MUSCLE and then manually corrected according to their secondary structure models [32,33]. Then, all these individual rDNA and PCG alignments without stop codons were concatenated into the following two datasets: PCGNTRNA and PCGNT12RNA (excluding the third codon positions of 13 PCGs). The substitution model and partitioning scheme of the two datasets were examined using IQ-TREE [34]. The details of best-fit models and partitioning schemes are summarized in Table S3. The BI and ML analyses were conducted using MrBayes 3.2.6 [35] and IQ-TREE, respectively. In BI analyses, two simultaneous runs of 5,000,000 generations were conducted, sampling every 100 generations, with a burnin of the first 1,040,000 generations. In ML analyses, node support values were assessed by 2000 ultrafast bootstrap replicates.

The ancestral state reconstruction for the gene order of three gene blocks was independently conducted using Mesquite 3.61 [36] with likelihood method (Markov k-state 1 parameter model) based on the ML phylogenetic tree.

## 3. Results and discussion

### 3.1. The General Features of Assassin Bugs Mitogenomes

**Figure 1:**
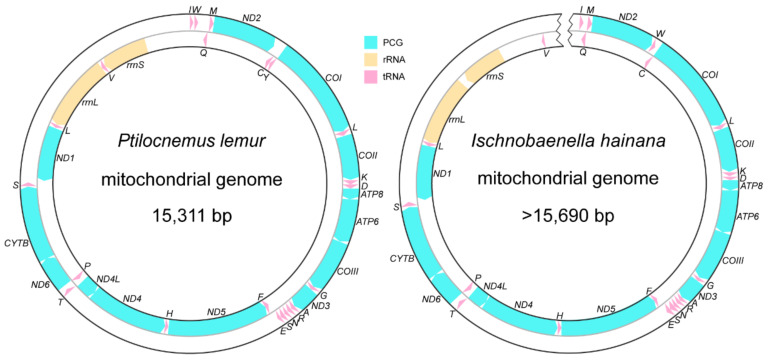
Circular maps of *P. lemur* and *I. hainana* mitogenomes. The transcriptional direction is denoted by arrows.

The complete mitogenome of *P. lemur* is 15,311 bp in size and contains 37 typical genes. The 15,690-bp nearly complete mitogenome of *I. hainana* contains only 36 genes, because the *trnY* could not be recognized around the conservative position between *ND2* and *COI* as well as other potential regions including the control region (CR). Both mitogenomes share the same strand distribution pattern of coding genes except for the undiscovered *trnY* of *I. hainana*: 23 genes are located on the majority strand; the other 14 genes are located on the minority strand (Figure 1). Although the size of reduviid mitogenomes ranges from 14,834 bp (*Physoderes impexa*) to 17,323 bp (*Triatoma migrans*) [37], the length of the coding region maintains a similar level, spanning the range between 14,424 bp (*Phymata americana*) [38] and 14,618 bp (*Phalantus geniculatus*) [17] with less than 200-bp differences. CR and fragments introduced by gene rearrangements are the main sources of genome size variability. Additionally, the genome size variation seems largely unrelated to reduviid phylogeny.

The mitogenomes of *P. lemur* and *I. hainana* have a relatively high A+T content (75.6%; 74.3%), a moderate A skew (0.15; 0.12), and a strong C skew (−0.25; −0.20) in the majority strand, which is similar to other reduviid mitogenomes [38]. The marked A+T bias of the base composition is also reflected in codon usage. For both mitogenomes, the synonymous codons ending with A/U always show a noticeably higher proportion than those with G/C. Meanwhile, the most prevalent codons (AUU, UUA, UUU, AUA, AAU, and UAU) consist solely of A and/or U (Table S4).

The evolutionary rate of 13 mitochondrial PCGs in Reduviidae was evaluated with the ka/ks ratio (Figure S1). All the average ka/ks ratios for each PCG are lower than 1—ranging from 0.087 (*COI*) to 0.805 (*ATP8*)—indicating that these PCGs evolved under the purifying selection, which is similar to other true bugs [15,39], while several exceptions (ka/ks > 1) detected in *ATP8* and *ND4L* reveal positive selection in the *ATP8* and/or *ND4L* of some assassin bugs, e.g., *P. lemur*. The average ka/ks ratios of *COI*, *COII*, *COIII*, and *CYTB* are clearly lower than those of the other nine genes, suggesting that cytochrome c oxidase and cytochrome c oxidoreductase were under stronger constraints.

### 3.2. Phylogenetic Analyses

**Figure 2:**
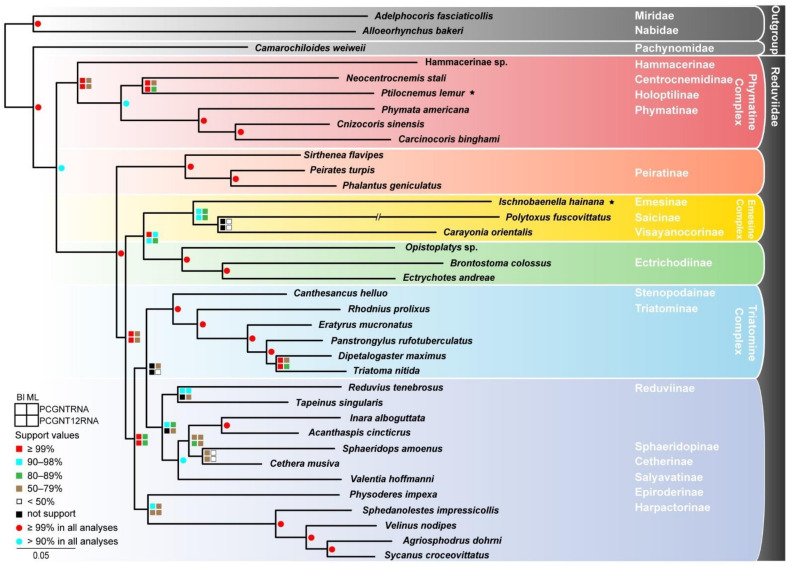
Phylogenetic tree of Reduviidae inferred from maximum-likelihood analyses based on PCGNTRNA and PCGNT12RNA datasets concatenated four rDNAs and 13 mitochondrial PCGs. Bootstrap values of maximum-likelihood analyses and posterior probabilities values of Bayesian analyses are labeled around each node. Asterisks denote new mitogenomes generated in this study.

With numerous efforts to investigate the phylogeny of Reduviidae during the past few decades, on the one hand some well-resolved phylogenetic results have been proposed [40,41,42,43,44,45,46], and on the other hand, there are still some conflicting evolutionary hypotheses in previous studies. Our phylogenetic analyses, based on concatenated mitochondrial and nuclear genes of 33 assassin bugs covering 17 subfamilies, achieved largely consistent topologies between the BI and ML methods (Figure 2 and Figure S2). The monophyletic Phymatine Complex *sensu lato* [44] is consistently recovered as the sister group to all the remaining reduviids (‘Higher Reduviidae’), which was also supported by previous molecular analyses [41,44]. The well-supported clade consisting of Centrocnemidinae, Holoptilinae, and Phymatinae underpins the reduction of ridges and teeth on the outside of the mandibles, the elongated pygophore, the processes on the endosomal struts of the phallus, and ventral glands as the synapomorphies of Phymatine Complex *sensu stricto* [40]. Within the ‘Higher Reduviidae’, the basal phylogenetic position of Peiratinae is constant across our analyses with high confidence. The subsequent split of a clade comprising Emesine Complex and Ectrichodiinae with the synapomorphies of the mostly membranous forewings and the absence of hooks or teeth on the ridges of the mandibles is in accordance with previous molecular analyses [41,45]. Additionally, the monophyly of this clade and the sister relationship between Emesine Complex and Ectrichodiinae were also supported by earlier morphological cladistic analyses [40] and molecular analyses [41]. Even though the limited Reduviinae samples were used in our phylogenetic reconstruction, the monophyly of Reduviinae is strongly rejected in all analyses. Two Triatomine Complex members, Stenopodainae and Triatominae, consistently form a clade, and Harpactorinae and Epiroderinae invariably show a close relationship as well, which is in agreement with the previous phylogenetic analyses [44] without regard to the unsampled Reduviinae lineages. The phylogenetic positions of Cetherinae, Salyavatinae, and Sphaeridopinae varied among our topologies, which may result from restricted sampling and incomplete data.

### 3.3. Gene Rearrangement in Heteroptera Mitogenomes

**Figure 3:**
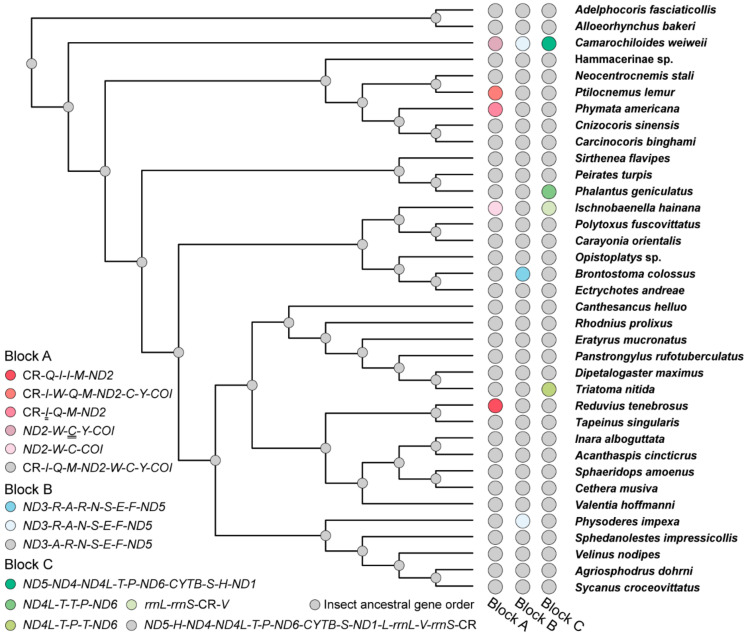
The ancestral state reconstruction of gene order for reduviid mitogenomes. The grey balls at nodes indicate all three gene blocks with the insect ancestral gene order. Genes marked with double underlines are absent in corresponding mitogenomes.

**Figure 4:**
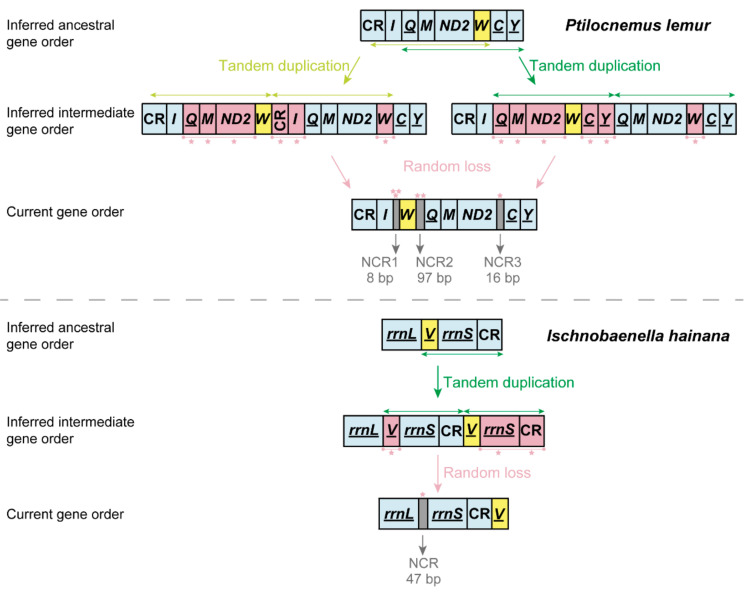
The inferred process of translocation of *trnW* (*P. lemur*) and *trnV* (*I. hainana*). CR: control region; NCR: non-coding region. Genes marked with underline are located on the minority strand.

The mitogenomes of *P. lemur* and *I. hainana* reported here show two novel gene orders that differ from all the reported gene orders of true bugs (Table 1). The ancestral state reconstruction of reduviid mitochondrial gene order demonstrates that the gene rearrangements of eight assassin bugs may well be derived independently from the insect ancestral gene order (Figure 3). For *P. lemur*, the *trnW* is translocated from the ancestral position between *ND2* and *trnC* to a new position between *trnI* and *trnQ*. For *I. hainana*, the *trnV* is relocated to the downstream of CR from the ancestral position between *rrnL* and *rrnS*; the *trnY* is not located between *trnC* and *COI*, which may be translocated to the unsequenced region between *trnV* and *trnI* or involved in gene loss. The translocation of *trnW* and *trnV* can be explained using the tandem duplication/random loss (TDRL) model [47,48] (Figure 4). With a common TDRL process, the tandem duplication of *trnQ*-*trnM*-*ND2*-*trnW* generated an intermediate gene order (*trnQ*-*trnM*-*ND2*-*trnW-trnQ*-*trnM*-*ND2*-*trnW*), and then the deletion or pseudogenization of *trnQ*, *trnM,* and *ND2* in the first copy and *trnW* in the second copy formed the current gene order of *P. lemur*. The first intergenic sequence (8 bp) between *trnI* and *trnW* and the third one (16 bp) between *ND2* and *trnC* can be well explained as the remnants of gene deletion or pseudogenization. However, the second intergenic sequence (97 bp) between *trnW* and *trnQ* cannot be introduced by the strict TDRL process of *trnQ*-*trnM*-*ND2*-*trnW*. To simplify the potential mechanism with the fewest TDRL steps, a broader boundary of tandem duplication (partial CR-*trnI*-*trnQ*-*trnM*-*ND2*-*trnW* or *trnQ*-*trnM*-*ND2*-*trnW*-*trnC*-*trnY*) may be used in the TDRL process. Under such scenarios, the deletion or pseudogenization of the partial CR-*trnI* in the second copy or *trnC*-*trnY* in the first copy resulted in the second 97-bp intergenic sequence. The translocation of *trnV* in the *I. hainana* mitogenome may have undergone the following scenarios: the sequence of *trnV*-*rrnS*-CR was tandemly duplicated to generate a repeating fragment (*trnV*-*rrnS*-CR-*trnV*-*rrnS*-CR), followed by the deletion or pseudogenization of *trnV* in the first copy and *rrnS* and CR in the second copy.

**Figure 5:**
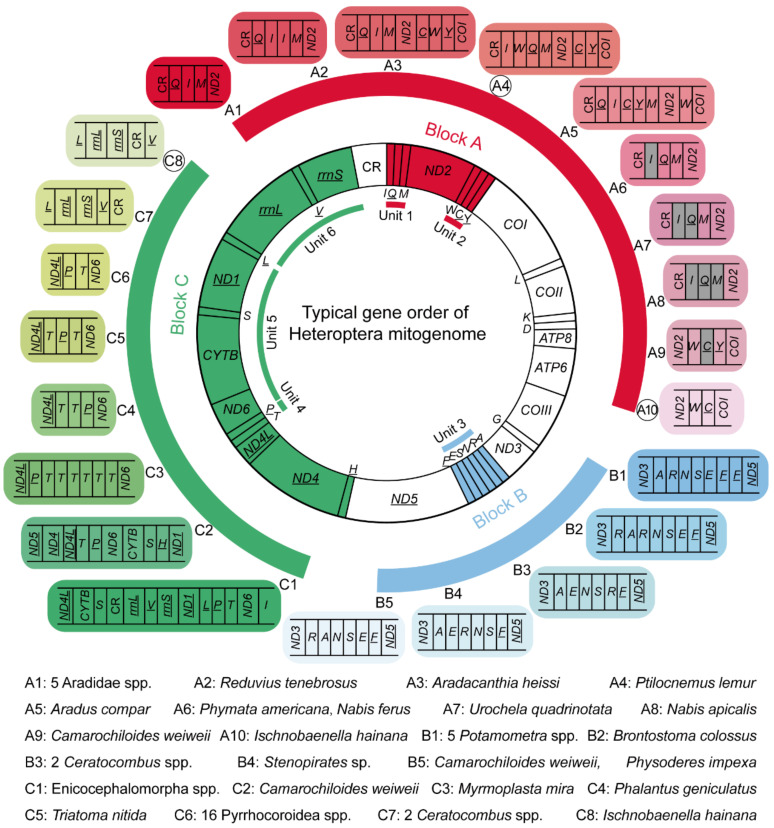
Typical gene order of heteropteran mitogenome and rearranged gene orders observed in true bugs. Blocks A–C denote mitochondrial genes involved in gene rearrangements. A1–A10, B1–B5, and C1–C8 indicate different gene orders for each block, of which A4, A10, and C8 with black circle are new gene rearrangements reported in this study. Units 1–6 indicate six divided rearrangement unit. Genes in gray are missing in corresponding mitogenomes. Genes marked with underline are located on the minority strand.

Gene rearrangement has long been considered a key aspect of mitogenome evolution. To better understand the evolution of heteropteran mitogenome, all 423 complete or partial mitogenomes were examined to recognize the diversity pattern of gene order. Heteropteran mitogenomes have a total of 21 different gene orders, among which the insect ancestral gene order is the ground pattern for Heteroptera. The rest of the 20 rearranged gene orders are found in 49 species from 11 families (Figure 5, Table 1 and Table S5). Overall, gene rearrangement has an uneven distribution pattern among true bugs. There is no known gene rearrangement in true aquatic bugs (Nepomorpha) and shore bugs (Leptopodomorpha) to date, while it exists, more or less, in the rest of the five infraorders (Enicocephalomorpha, Dipsocoromorpha, Gerromorpha, Cimicomorpha, and Pentatomomorpha). The frequent occurrence of gene rearrangement has been often considered to be associated with unusual lifestyles, such as parasitic wasps [49,50]. However, no evident concentration of gene rearrangement is closely related to any type of unusual feeding habit or lifestyle in true bugs, e.g., hematophagous lineages, indicating such relaxed association between feeding habit or lifestyle and mitochondrial gene arrangement is not present in true bugs.

Heteropteran gene rearrangements comprise three rearrangement types, i.e., gene translocation, gene duplication, and gene loss (Table 1). Among which, gene translocation involves at least ten tRNA genes and two PCGs, while gene duplication and gene loss occur in only four tRNA genes, respectively. Therefore, tRNA gene translocation is the main form of heteropteran gene rearrangement. Mapping all the rearranged genes to the ground gene order of true bugs, their locations are concentrated in three gene blocks (Figure 5). Block A (genes between CR and *COI*) displays the highest rearrangement diversity with ten different orders, and block B (tRNA gene cluster between *ND3* and *ND5*) contains only five variants. Block C, with the largest number of component genes (between *ND5* and CR), shows a relatively high rearrangement diversity. These three blocks were further divided into six rearrangement units (unit 1: *trnI*-*trnQ*-*trnM*, unit 2: *trnW*-*trnC*-*trnY*, unit 3: *trnA*-*trnR*-*trnN*-*trnS*-*trnE*-*trnF*, unit 4: *trnT*-*trnP*, unit 5: *ND6*-*CYTB*-*trnS*-*ND1*, and unit 6: *rrnL*-*trnV*-*rrnS*), with the aim of investigating the rearrangement hotspot and their distribution bias in true bugs. Comparing all the rearrangement units, the tRNA gene cluster *trnI*-*trnQ*-*trnM* shows a higher rearrangement diversity than the other units, and the tRNA gene clusters *trnA*-*trnR*-*trnN*-*trnS*-*trnE*-*trnF* and *trnT*-*trnP* show the highest taxon coverage of rearrangement, with five families, respectively. The rearrangement unit richness among the families (Table 2) reveals that Reduviidae encompass all the rearrangement units except for the *ND6*-*CYTB*-*trnS*-*ND1* unit. Moreover, the highest rearrangement richness of most units can be constantly found in Reduviidae, and the rearrangement richness of the whole mitogenome reached up to 40.0% in Reduviidae, suggesting Reduviidae is a ‘hot-spot group’ of gene rearrangement in Heteroptera.

Most gene rearrangements of insect mitogenomes can be clarified using TDRL or/and recombination models [51]. Owing to a lack of gene inversion and long-range translocation, the TDRL model applies to almost all the gene rearrangements of heteropteran mitogenomes. Apart from the TDRL model, another mechanism consisting of the TDRL model and tRNA remolding was reported to explain the gene rearrangement of the *Reduvius tenebrosus* mitogenome (Reduviidae) [16]. Mitochondrial gene loss is uncommon for most insect orders, but this phenomenon has been reported in several insect and other metazoan lineages, such as the absence of *trnI* in *Aleurocanthus camelliae* (Aleyrodidae) [52], *ATP8* in Neodermata (Platyhelminthes) [53], and 23 genes (*ATP6*, *ATP8*, and 21 tRNA genes) in some *Sagitta* spp. (Chaetognatha) [54]. All the missing genes in heteropteran mitogenomes are tRNA genes located in block A. The high frequency of gene rearrangement in this block, especially for *trnI*-*trnQ*-*trnM* (unit 1), may provide the probability of tRNA gene loss in a TDRL process. Additionally, these missing mitochondrion-encoded tRNAs can be functionally replaced by the corresponding nucleus-encoded tRNAs [55] or RNA editing of the mitochondrion-encoded tRNA [56]. Indeed, the reported heteropteran gene rearrangements are not involved in recombination, but this does not mean heteropteran mitogenomes without recombination. Actually, repeated sequences located far away from each other or separated inverted-repeated sequences in non-coding regions [21] imply that recombination events may exist in heteropteran mitogenomes.

From an evolutionary point of view, we assessed the gene rearrangements as potential synapomorphy for each lineage (Table 1). The gene order C1 in block C is present in all the sequenced mitogenomes of the unique-headed bugs, indicating that this rearrangement event may occur at least in the most recent common ancestor of Aenictopecheidae and Enicocephalidae, and the gene order C1 has been retained during the subsequent evolution of unique-headed bugs as a potential synapomorphy for this infraorder. Another gene rearrangement B4 should be obtained during the evolution of *Stenopirates* sp. The gene order A1 can be regarded as a synapomorphy for Aradidae, kept in Aneurinae, Carventinae, and Mezirinae. The other two gene rearrangements, A3 (Aradidae-Calisiinae) and A5 (Aradidae-Aradinae), independently evolved from A1 [57]. The gene rearrangement C6 (*trnP*-*trnT*) exists in all Pyrrhocoroidea spp. as a potential synapomorphy for this superfamily. Two *Ceratocombus* spp. (Ceratocombidae) share the same gene rearrangements B3 (*trnA*-*trnE*-*trnN*-*trnS*-*trnR*-*trnF*) and C7 (*rrnL*-*rrnS*-*trnV*-CR) as the potential synapomorphies for this genus. Compared with the heteropteran clades mentioned above, eight rearranged gene orders scattering in eight reduviid subfamilies evolved independently in each corresponding lineage. Additionally, there is no closely related genus or species with the gene rearrangement within these subfamilies as yet. These temporal and lineage distributions of gene rearrangements draw the evolutionary history of gene arrangement for the Heteroptera mitogenome. It should be noted that the same gene rearrangement can be shared by different species with distant relationships, e.g., both *Nabis ferus* (Nabidae) and *P. americana* (Reduviidae) lost the *trnI* (Figure 5, Table 1), indicating the convergence of gene rearrangement with misleading phylogenetic signal.

## 4. Conclusions

In this study, the mitogenomes of two assassin bugs *P. lemur* and *I. hainana*, the first representatives of Holoptilinae and Emesinae, respectively, were determined. Both mitogenomes contain tRNA gene translocation resulting in two novel gene orders. The possible rearrangement processes were inferred based on the TDRL model. Furthermore, we summarized all the gene rearrangements of the heteropteran mitogenomes involved in three gene blocks and identified the tRNA gene cluster *trnI*-*trnQ*-*trnM* (unit 1 in block A) as the rearrangement hotspot of true bugs. Reduviids with the most diverse gene orders for most rearrangement units are the ‘hot-spot group’ of mitochondrial gene rearrangement in Heteroptera. Finally, phylogenetic reconstructions based on 13 mitochondrial PCGs and four rDNAs provide a relatively robust phylogenetic framework of Reduviidae, especially for the deep nodes, which reinforces the understanding of the phylogenetic relationships of reduviid basal lineages.

## Figures and Tables

**Table 1:** Gene rearrangements in heteropteran mitogenomes.

Infraorder	Family (Species Number)	Rearranged Gene Order	Potential Synapomorphy	Rearrangement Type
Enicocephalomorpha	Aenictopecheidae (1)	*ND4L*-*CYTB*-*S*-CR-*rrnL*-*V*-*rrnS*-*ND1*-*L*-*P*-*T*-*ND6-I*	infraorder	gene translocation
	Enicocephalidae (4)			
	Enicocephalidae (1)	*A*-*E*-*R*-*N*-*S*-*F*-//-*ND4L*-*CYTB*-*S*-CR-*rrnL*-*V*-*rrnS*-*ND1*-*L*-*P*-*T*-*ND6-I*	/	gene translocation
Dipsocoromorpha	Ceratocombidae (2)	*A*-*E*-*N*-*S*-*R*-*F*-//-*rrnL*-*rrnS*-*V*-CR	genus	gene translocation
Gerromorpha	Gerridae (5)	*A-R-N-S-E-F-F*	genus	gene duplication
Cimicomorpha	Nabidae (1)	CR-*I*-*Q*-*M*-*ND2*	/	gene loss
	Nabidae (1)	CR-*I*-*Q*-*M*-*ND2*	/	gene loss
	Pachynomidae (1)	*ND2*-*W*-*C*-*Y*-//-*R*-*A*-*N*-*S*-*E*-*F*-//-*CYTB*-*S*-*H*-*ND1*	/	gene loss, gene translocation
	Reduviidae (1)	CR-*Q*-*I*-*I*-*M*-*ND2*	/	gene duplication, gene translocation
	Reduviidae (1)	CR-*I*-*W*-*Q*-*M*-*ND2*-*C*-*Y*-*COI*	/	gene translocation
	Reduviidae (1)	*ND2*-*W*-*C*-*COI*-//-*rrnL*-*rrnS*-CR-*V*	/	gene translocation
	Reduviidae (1)	CR-*I*-*Q*-*M*-*ND2*	/	gene loss
	Reduviidae (1)	*R*-*A*-*R*-*N*-*S*-*E*-*F*	/	gene duplication, gene translocation
	Reduviidae (1)	*R*-*A*-*N*-*S*-*E*-*F*	/	gene translocation
	Reduviidae (1)	*ND4L*-*T*-*T*-*P*-*ND6*	/	gene duplication
	Reduviidae (1)	*ND4L*-*T*-*P*-*T*-*ND6*	/	gene duplication, gene translocation
Pentatomomorpha	Aradidae (5)	CR-*Q*-*I*-*M*-*ND2*	family	gene translocation
	Aradidae (1)	CR-*Q*-*I*-*M*-*ND2*-*C*-*W*-*Y*-*COI*	/	gene translocation
	Aradidae (1)	CR-*Q*-*I*-*C*-*Y*-*M*-*ND2*-*W*-*COI*	/	gene translocation
	Largidae (4)	*ND4L*-*P*-*T*-*ND6*	superfamily	gene translocation
	Pyrrhocoridae (12)			
	Pyrrhocoridae (1)	*ND4L*-*P*-*T*-*T*-*T*-*T*-*T*-*T*-*ND6*	/	gene duplication, gene translocation
	Urostylididae (1)	CR-*I*-*Q*-*M*-*ND2*	/	gene loss

Note: genes marked with underline are located on the minority strand; genes marked with double underlines are absent in corresponding mitogenomes.

**Table 2:** Gene rearrangement richness of rearrangement units and whole mitogenome for heteropteran families.

Family	*I-Q-M* (Block A)	*W-C-Y* (Block A)	*A-R-N-S-E-F* (Block B)	*T-P* (Block C)	*ND6-CYTB-S-ND1* (Block C)	*rrnL-V-rrnS* (Block C)	Whole Mitogenome
Aenictopecheidae	0	0	0	25.0%	**50.0%**	0	5.0%
Enicocephalidae	0	0	20.0%	25.0%	**50.0%**	0	10.0%
Ceratocombidae	0	0	20.0%	0	0	**50.0%**	5.0%
Gerridae	0	0	20.0%	0	0	0.0%	5.0%
Nabidae	28.6%	0	0	0	0	0	10.0%
Pachynomidae	0	20.0%	20.0%	0	**50.0%**	0	5.0%
Reduviidae	**42.9%**	**40.0%**	**40.0%**	**50.0%**	0	**50.0%**	**40.0%**
Aradidae	28.6%	**40.0%**	0	0	0	0	15.0%
Largidae	0	0	0	25.0%	0	0	5.0%
Pyrrhocoridae	0	0	0	**50.0%**	0	0	10.0%
Urostylididae	14.3%	0	0	0	0	0	5.0%

## Data Availability

Annotated mitogenomes were deposited into GenBank under the accession numbers: MW540749 and MW619686.

## Supplementary Materials

The following are available online at https://www.mdpi.com/article/10.3390/genes12081134/s1, Figure S1: Evolutionary rate of each mitochondrial PCG of Reduviidae, Figure S2: Phylogenetic trees of Reduviidae inferred from Bayesian analyses based on PCGNTRNA and PCGNT12RNA. Posterior probabilities values are labeled around each node, Table S1: The NCBI accession numbers of mitogenomes used to calculate the Ka/Ks ratios, Table S2: The NCBI accession numbers of sequences used in phylogenetic analyses, Table S3: The partitioning schemes and models used for phylogenetic analyses, Table S4: Codon usage pattern of *Ptilocnemus lemur* and *Ischnobaenella hainana* mitogenomes, Table S5: The NCBI accession numbers of true bugs mitogenomes with gene rearrangement.
