# Supplementary figures and images for: Mitochondrial Genomes from Two Specialized Subfamilies of Reduviidae (Insecta: Hemiptera) Reveal Novel Gene Rearrangements of True Bugs
Source: Genes (Basel). 2021 Jul 26;12(8):1134. doi: 10.3390/genes12081134 (PMC8392325; doi:10.3390/genes12081134)

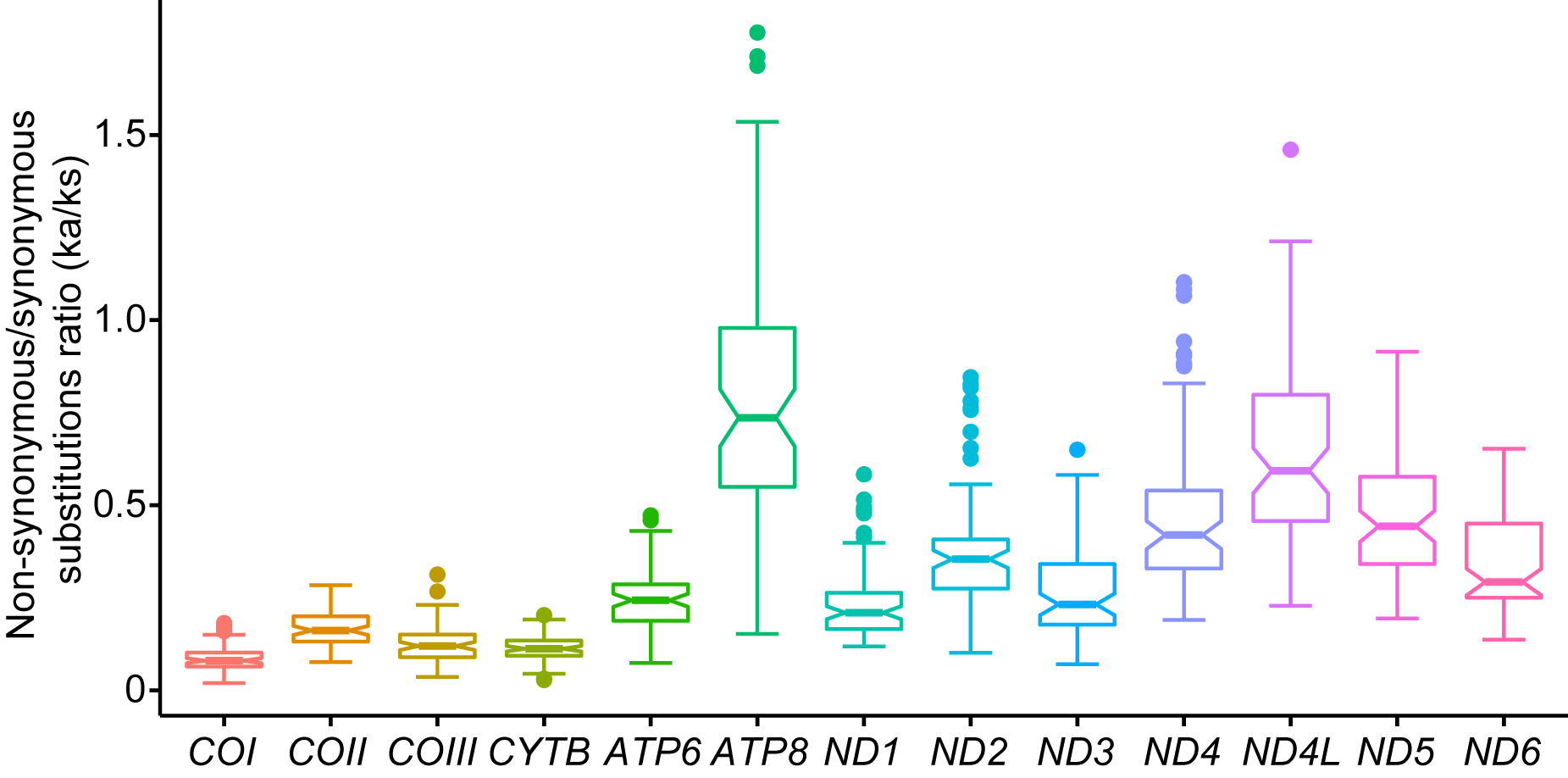

Supplement: Supplementary file 1 [file genes-12-01134-s001.zip › Supplementary Files-20210726/Figure S1.tif]

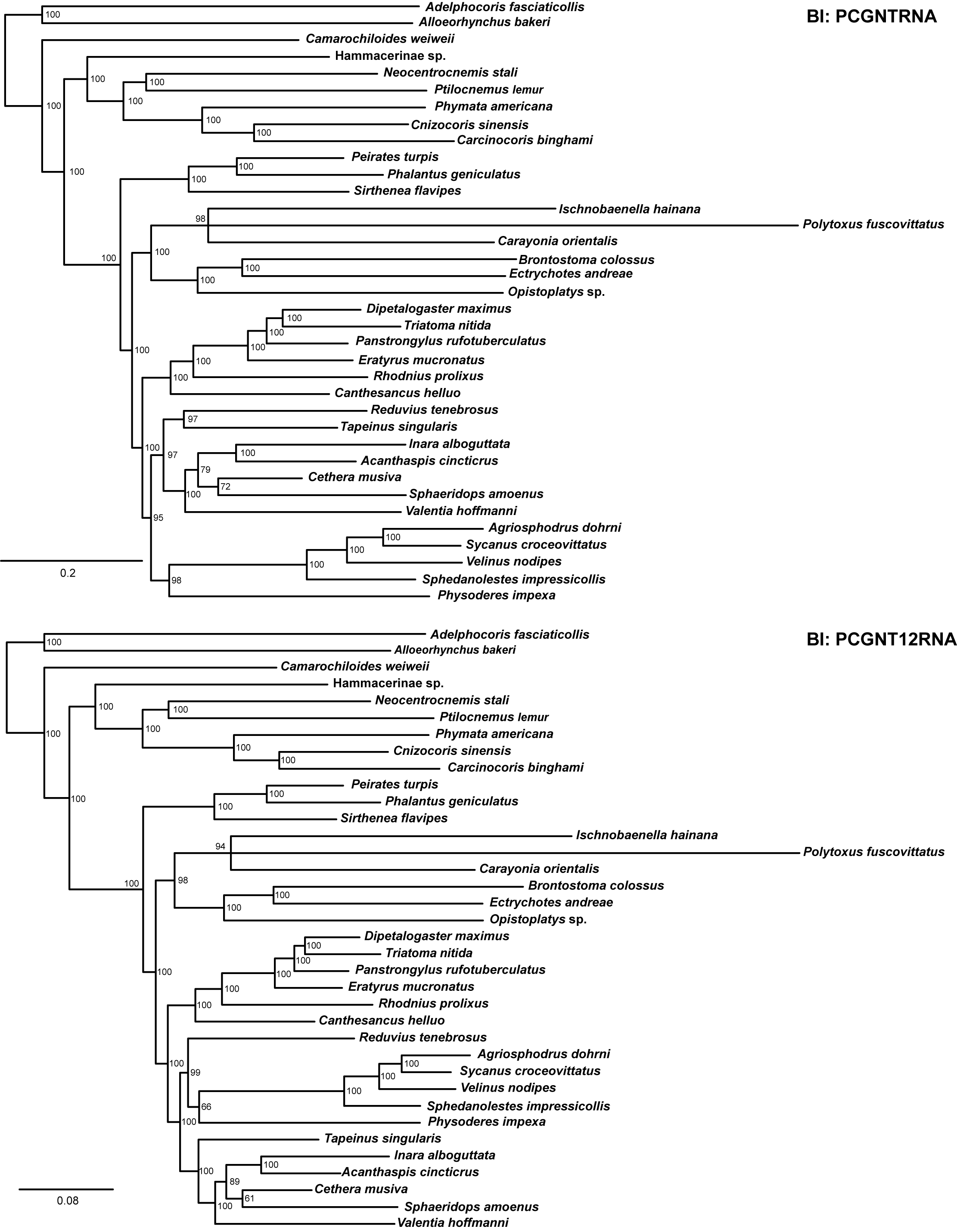

Supplement: Supplementary file 1 [file genes-12-01134-s001.zip › Supplementary Files-20210726/Figure S2.tif]
